# Climatic changes: knowledge and adaptation behavior to heat-related illness among solid waste disposal workers

**DOI:** 10.1186/s42506-024-00155-x

**Published:** 2024-05-06

**Authors:** Raghda A. Elshamy, Afaf M. Eladl, Mona F. Zaitoun

**Affiliations:** Department of Community, Environmental and Occupational Medicine, Faculty of Medicine, Zagazig University, Zagazig, Egypt

**Keywords:** Climatic changes, Knowledge, Adaptation behavior, Heat-related illness, Solid waste disposal workers

## Abstract

**Background:**

Earth’s climate changes are progressing at an alarming rate. One of the most severe effects of climate change is extreme heat. This study aimed to assess knowledge and adaptation behavior to heat-related illness (HRI) among solid waste disposal workers in the 10th of Ramadan City, Egypt, and to study the predictors for their knowledge and adaptation behavior.

**Methods:**

An exploratory cross-sectional study was conducted on 220 solid waste disposal workers. A structured interview questionnaire was used to assess the studied workers’ sociodemographic and occupational characteristics, heat exposure risk, and their knowledge, and adaptation behavior.

**Results:**

The results showed that 40% and 30% of participants had adequate levels of total knowledge and adaptation behavior, respectively. There was a statistically significant relationship between workers’ knowledge and both age and education. There was a statistically significant relationship between workers’ adaptation behavior and age, duration of employment, working hours, and education. A binary logistic regression for significant predictors of knowledge and adaptation behavior showed that age and education were the most significant predictors.

**Conclusion:**

Solid waste disposal workers were at high risk of HRI due to their low levels of knowledge and adaptation behavior regarding HRI. Educational health programs that guide workers to follow healthy behaviors and prevent HRI are recommended.

## Introduction

Workers who remain outdoors for a long time, such as agriculture, construction, firefighting, manufacturing, and collecting solid waste, are considered outdoor workers [1]. According to scientific data, increases in greenhouse gases are the principal cause of the earth’s climate change at an alarming rate [2]. One of the most severe effects of climate change is extreme heat [1]. Representative concentration pathways (RCPs) predicted that concentrations of greenhouse gases in the atmosphere will change in the future with the worst scenario predicting that the average global temperature of the human habitation environment will increase by 7.5 °C in 2070 compared to the preindustrial period 300 years ago [3].

Temperatures in Egypt have already increased over the past decades (0.53 °C per decade over the last 30 years). By mid-century, temperatures are expected to increase between 1.5 and 3 °C. Heat waves will increase in their severity, frequency, and duration, with an average of 40 additional days of extremely hot days per year [4].

Extreme heat exposure raises morbidity and mortality rates [5]. In tropical countries, climatic change and a rise in intense heat threaten occupational health and labor productivity [6]. Heat can impede economic growth and exacerbate poverty [7]

It is expected that there will be hundreds more cases of heat-related illness (HRI) annually by 2030 and 1000 to 2000 more cases annually by 2060. Furthermore, it is predicted that the death rate from heat stress will rise by nearly 300% by the year 2100, assuming an increase in average global temperatures of 3 °C [8].

Numerous studies have shown that working in hot weather without the proper safety precautions, particularly outside, can cause heat-related symptoms and illnesses ranging from just mild dehydration and heat rash to life-threatening heat stroke [9].

Many recent epidemiological studies have provided evidence of the association between heat exposure and the risk of accidents at work, and this phenomenon can be explained by a decrease in cognitive performance in people who work in hot environment [10].

Waste disposal workers are isolated, vulnerable, and neglected workers, and they are frequently in significant danger of occupational health problems, particularly in the underdeveloped world. Little is known about their knowledge and adaptation behavior regarding HRI. This study aimed to assess the knowledge and adaptation behavior to HRI among solid waste disposal workers and to study the predictors for their knowledge and adaptation behavior.

## Methods

### Study design

An exploratory cross-sectional study was conducted among solid waste disposal workers during the summer season from June to September 2022 at 10th of Ramadan City.

### Study area

The city of 10th of Ramadan is an industrial city, considered the oldest and largest industrial city in Egypt. It was established by the presidential decree (249/1977). The full-time residential population of the city is about 500,000. Köppen–Geiger climate classification system classifies its climate as a hot desert with an average temperature of 70 °F in summer [11]. Solid waste collection is a big concern for waste management. Waste is collected and transferred to the existing public dumpsite, located 6 km south of the city, so many workers are at high risk of exposure to high temperatures with the occurrence of HRI [12].

### Study population

The study population was solid waste disposal workers above 18 years old. They were working in collecting, transporting, and separating solid waste at the time of the study and they reported that they were not suffering from any chronic disease.

### Sample size and sampling technique

Open Epi was used to calculate the required sample size. Using the following formula (*n* = [DEFF × Np (1−*p*)]/[(d2/Z21−/2 × (*N*−1) +*p* × (1−*p*)]) *n* = required sample size, ∝/2 = 2.57 (99% CI), *P* is the prevalence of the outcome (percent of workers’ knowledge about HRI was 21% [13]; *N* is the population size (for a finite population correction factor or fpc = 600); d is the margin of error, 0.05; DEFF is the design effect (for cluster surveys, here assumed to be 1). With a precision of 5%, a 95% confidence interval, and 80%, the minimal required size should account for 184 participants. Adding 20% to compensate for potential non-response, the minimal sample size was estimated to be 220 participants. Five residential districts were selected randomly at 10th of Ramadan City (Alandalose, 25th, 27th, 34th, and care house districts). In each selected district, workers were selected by a simple random sampling technique using a workers’ list. Consecutive samples were collected until the required sample size was fulfilled.

### Tools of data collection

All workers were asked questions adapted from previous studies [9, 13–15]. The questions covered the following sections

- Section I: Sociodemographic and occupational characteristics of the studied workers: age, sex, residence, marital status, educational level, smoking status, type of task, duration of employment, and number of worked hours/day.
- Section II: This section comprised eight questions aimed at assessing the perception of heat exposure risks in the workplace. These questions covered whether the worker was aware of heat-related illness (HRI), the source of their knowledge about HRI, the presence of rising temperatures at the workplace, any observed increase in mortality during summer, the perceived intensity of summer temperatures, the incidence of illness among workers during the summer, and whether workers use personal protective equipment (PPE) for prolonged periods in high temperatures.
- Section III: This section assessed their knowledge regarding HRI and was comprised of 17 questions (8 yes, no, or do not know items and 9 multiple-choice items). Questions covered general knowledge, clinical picture, and prevention of HRI. According to Sraku–Lartey et al. [13], participants received one point for answering each correct item, and incorrect answers received zero points. The score range for the knowledge was 0–17. An adequate knowledge level was deemed present when the total score was equal to or above 50% (8 points).
- Section IV: This section assessed their adaptation behavior regarding HRI, comprising 10 objective questions (5 yes, no, or Do not know items and 5 multiple-choice items). Questions covered prevention and emergency aids of HRI. According to Sraku–Lartey et al. [13], participants received one point for answering each correct item, and incorrect answers received zero points. The score range for adaptation behavior was 0–10. Adequate adaptation behavior was deemed present when the total score was equal to or above 50% (5 points).

### Pilot test

One month before the start of this study, a pilot study was conducted to test any data collection difficulties and to estimate the time needed for data collection. Linguistic experts translated the questionnaire into Arabic and then back to English. Results showed that all the items were internally consistent and reliable. Cronbach’s alpha for knowledge questions was 0.82, and for questions of adaptation behavior was 0.74. Minor modification of the questionnaire was done, for example, the question “Sweating is instrumental in dissipating excessive heat?” was modified to “Sweating is useful for reducing body temperature?” and the question “What are the environmental implications of climate change?” was modified to “What are the effects of climate change on the environment?” The questionnaire took nearly 20 min to complete with a 100% response rate. The final analysis did not include any of the pilot data.

### Data management

Data was coded, entered, and analyzed using Microsoft Excel software. Data was then imported into Statistical Package for the Social Sciences (SPSS version 26.0) software for analysis. According to the data type, qualitative data was represented as number and percentage; quantitative data was represented by mean ± SD. The relation between either knowledge or adaptation behavior and the role of some sociodemographic or occupational factors was assessed using Pearson’s chi-square test and *t*-test. Binary logistic regression assessed significant predictors for knowledge and adaptation behavior. The significance of the obtained results was judged at the 5% level.

## Results

Results showed that the average age of the studied workers was 37 ± 7.8 years. All of them were males. Fifty percent were waste collectors. The mean ± SD duration of employment was 13.5 ± 5.7 years with 8.9 ± 0.95 h worked per day (Table 1).

**Table 1:** Sociodemographic and occupational data among solid waste disposal workers at 10th of Ramadan City, Egypt (*n* = 220)

Variable	No. (%)
**Age (years)**	Mean ± SD, 37 ± 7.8 Range, 25–50
**Sex**	
Male	220 (100)
**Residence**	
Urban	132 (60)
Rural	88 (40)
**Marital status**	
Married	132 (60)
Not married	88 (40)
**Educational level**	
Primary	66 (30)
Preparatory	22 (10)
Secondary	110 (50)
University	22 (10)
**Smoking**	
Never smoked before	154 (70)
Ex-smoker	44 (20)
Current smoker (for at least 1 year)	22 (10)
**Occupation**	
**1-Type of task**	
Collector Driver	110 (50) 66 (30)
Separator	44 (20)
**2-Duration of employment (years)**	Mean ± SD, 13.5 ± 5.7 Range, 7–23
**3-No. of hours worked/day**	Mean ± SD, 8.9 ± 0.95 Range, 8–10

Regarding the perception of heat exposure risks, more than one-third of the workers had heard about the concept of climate change or heat stress, with about one-third hearing about it from the media. About two-thirds of them reported that temperatures had increased significantly in the last 2 years. One-third reported that heat events contribute to higher mortality and that illness rates increase more during summer than winter. The majority of them reported that the conditions of their workplaces in summer are hotter and more humid and that they spent long hours under direct sunlight. Half of them got sick between June and November while working in the heat. More than half of them used personal protective equipment (non-tabulated results).

Regarding the knowledge of the workers, about two-thirds reported that wearing thicker working clothes does not help maintain a low body temperature. Half of them reported that sweating helps reduce the body temperature and had adequate knowledge regarding the causes of climate change or heat stress. About one-third had adequate knowledge about the effects of climate change on the environment, and a similar proportion had adequate knowledge about types of heat-related illnesses. Twenty percent reported that heat stroke is the most serious heat-related illness (Table 2). The results also showed that about two-thirds had adequate knowledge about the symptoms of heat exhaustion. More than two-thirds had adequate knowledge about the symptoms of a heat stroke. More than half reported that fainting and collapse could be due to heat-related illnesses. Only 10% had adequate knowledge about factors that increase the risk of a heat-related disease (Table 2).

**Table 2:** Knowledge assessment items regarding HRI among solid waste disposal workers at 10th of Ramadan City, Egypt (*n* = 220)

Variable and its correct answers	No. (%)
1. Body temperature is normally not higher than 38 °C? (yes)	44 (20)
2. Wearing thicker working clothes is not useful to maintain low body temperature? (yes)	132 (60)
3. Wearing thinner working clothes is useful to maintain low body temperature? (yes)	88 (40)
4. Sweating is useful for reducing body temperature? (yes)	110 (50)
5. Not only children and elders are at health risk in case of high temperatures and heat illness. (yes)	22 (10)
#6. What do you think causes climate change or heat stress? (pollution, naturally)	110 (50)
#7. What are the effects of climate change on the environment? (wildfires, destroying forests, increase in pests and diseases, land degradation)	66 (30)
#8. What are the types of heat-related illnesses? (heat cramps, heat exhaustion, heat stroke, heat syncope)	66 (30)
#9. Which type of heat-related illnesses are the most serious? (heat stroke)	44 (20)
10. Is there a difference between heat cramps, heat exhaustion, and heat stroke? (yes)	22 (10)
#11. What are the symptoms of heat cramps? (sweating, muscle ache, fever, cramps)	110 (50)
#12. What are the symptoms of heat exhaustion? (headache, sweating, fever, nausea and vomiting, dizziness, cold skin)	132 (60)
#13. What are the symptoms of heat stroke? (confusion, fever, nausea and vomiting, difficulty breathing, fainting, hot skin)	154 (70)
14. Could fainting and collapse be due to heat-related illnesses? (yes)	132 (60)
#15. Please select the symptoms or signs of heat-related illnesses that you consider to be severe. (fainting)	88 (40)
16. Can heat-related illnesses cause a rapid loss of the victim’s life? (yes)	44 (20)
#17. Which of the following factors increases the risk of heat-related disease? (aging, overweight, alcohol, not enough fluid intake)	22 (10)

**#**Multiple choice questions

Regarding adaptation behavior, more than one-third had adequate heat prevention measures adopted in the workplace, reported that they would seek a doctor if they suffer from any heat-related illness, and had adequate adaptation behavior if a worker gets heat stroke (Table 3).

**Table 3:** Adaptation behavior assessment items regarding HRI among solid waste disposal workers at 10th of Ramadan City, Egypt (*n* = 220)

Variable and its correct answers	No. (%)
#1. What are heat prevention measures which can be adopted in the workplace? (provision of cool drinking water at the workplace, rescheduling work time, shady rest areas, stopping work if the temperature exceeds 40, wearing a hat, training sessions on how to adapt)	88 (40)
2. Can constructing illegal shelters made from cardboard boxes, canvas, and plastic sheeting protect from heat-related illness? (no)	44 (20)
#3. If you suffer from any heat-related illness, what will you do? (seek a doctor)	88 (40)
4. Do you think heat illness is managed by transferring the victim to a cool environment, drinking fluids, and applying cool water, ice packs, and fans? (yes)	22 (10)
5. When a heat stroke is suspected, should you first transfer the victim to a cool environment and then ask for an ambulance? (yes)	33 (15)
6. Which drink would you prefer for a heat victim? (water or ORS solution)	66 (30)
7. When do you take water at a hot workplace? (every hour)	44 (20)
#8. What will you do if a worker gets heat cramps? (give oral rehydration solution (ORS))	44 (20)
#9. What will you do if a worker gets heat exhaustion? (call emergency, move to cool place, loosen clothes, put a wet cloth on the body, sip water, fanning)	44 (20)
#10. What will you do if a worker gets a heat stroke? (call emergency, move to cool place, loosen clothes, put a wet cloth on the body, sip water, fanning)	88 (40)

**#**Multiple choice questions

**Fig. 1:**
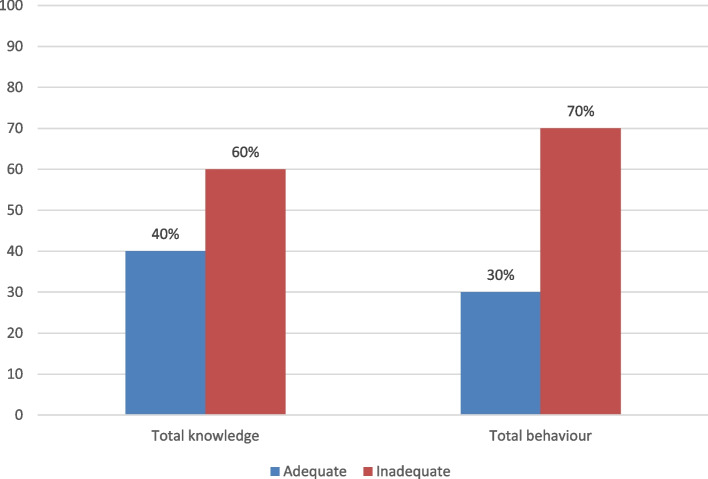
Level of total knowledge and adaptation behavior among solid waste disposal workers at the 10th of Ramadan City (*n* = 220)

 The total knowledge and adaptation behavior scores were adequate in 40% and 30% of the sample, respectively (Fig. 1).

There was a statistically significant relationship between knowledge and both age and education. There was a statistically significant relationship between adaptation behavior, age, duration of employment, working hours, and education (Table 4).

**Table 4:** Relationship between some sociodemographic and occupational data, knowledge, and adaptation behavior among solid waste disposal workers

Variable	Knowledge		*P*-value	Adaptation		*P*-value
	Adequate (*n* = 88)	Inadequate (*n* = 132)		Adequate (*n* = 66)	Inadequate (*n* = 154)	
**Age (years)**						
**Mean ± SD**	32 ± 4.4	40.4 ± 7.7	< 0.001*	32 ± 5.1	39.1 ± 7.7	< 0.001*
**Range**	25–37	28–50		27–37	28–50	
**Duration of employment (years)**						
**Mean ± SD**	13.3 ± 6.5	13.7 ± 5.1	0.596	15 ± 6.6	12.9 ± 5.2	0.010*
**Range**	7–23	8–20		7–23	8–20	
**Working hours**						
**Mean ± SD**	9 ± 1	8.8 ± 0.9	0.201	8.7 ± 0.95	9 ± 0.93	0.016*
**Range**	8–10	8–10		8–10	8–10	
**Education**						
**Primary**	22 (25)	44 (33.3)	<0.001*	22 (33.3)	44 (28.6)	<0.001*
**Preparatory**	0 (0)	22 (16.7)		0 (0)	22 (14.3)	
**Secondary**	44 (50)	66 (50)		44 (66.7)	66 (42.9)	
**University**	22 (25)	0 (0)		0 (0)	22 (14.3)	

P* significant at level < 0.05

A binary logistic regression for significant predictors of knowledge and adaptation behavior among the studied workers showed that age and education were their most significant predictors (Table 5).

**Table 5:** Logistic regression for significant predictors of knowledge and adaptation behavior among solid waste disposal workers

Variable	B	S.E	Wald	O.R (95% C.I)	*P*-value
**Knowledge**					
Age	0.317	0.047	44.6	1.37 (1.25–1.51)	< 0.001*
Education	0.53	0.79	12.18	1.98 (1.05–4.15)	< 0.001*
**Adaptation behavior**					
Age	0.316	0.046	44.5	1.36 (1.25–1.51)	< 0.001*
Duration of employment	1.64	0.49	3.99	2.69 (1.06–2.75)	0.91
Working hours	0.79	0.22	2.17	2.04 (1.89–4.65)	0.35
Education	0.54	0.78	12.19	1.99 (1.05–4.15)	< 0.001*

*B* beta, *SE* standard error, *OR* odds ratio. P* significant at level < 0.05

Duration of employment and working hours were not included under the knowledge item because they were not significantly related to knowledge

## Discussion

This study revealed inadequate knowledge and adaptation behavior among the studied population, with relatively high knowledge gaps. Age and education were the most significant predictors of knowledge and adaptation behavior.

The term climate change was understood by less than half of the study’s participants; these results were similar to the study in the Offinso municipality, Ghana, where only 31.9%of respondents had heard about it [13]. Media and the internet were the primary and most common sources of heat-related information for solid waste disposal workers, as observed in similar studies from China, Ghana, and South Australia [9, 13, 16].

On the other hand, a survey study among health and safety representatives in Italy revealed adequate high knowledge about climate change when healthcare providers were identified as the primary information source by respondents (34.4%), followed by professional courses (28.2%), and conventional media (14.3%) [15]. Media and mobile apps can be used as an opportunity for information and online training. A USA study found that smartphone/tablet applications and online training were occupational workers’ preferred heat stress training methods [17].

It should be stressed that while conventional media and new media frequently emphasize the emotional aspects of climate change, they occasionally raise the concerns of their audience; more scientifically accurate information sources such as professional courses and healthcare professionals usually address such a phenomenon through a rigid purely rational explanation that may be mistakenly perceived as condescending [18]. To avoid this, the Occupational Safety and Health Administration (OSHA) and the National Institute of Occupational Safety and Health (NIOSH) in the USA jointly developed a mobile app that can be a helpful tool for determining how hot it feels during the day and using that information to plan outside job activities. It has a real-time heat index and hourly forecasts specific to worker location. It also provides occupational safety and health recommendations [19].

The interest in the impact of heat-related events on workers’ health and safety has recently increased. Evidence suggests that scorching weather contributes to excess morbidity and mortality, particularly among older aged individuals and patients undergoing pharmacologic treatment for non-communicable diseases [20]

Half of the participants in this study got sick between June and November while working in the heat. This illness rate is lower than the rate reported by two studies of outdoor workers in Zimbabwe [14] and the USA [21] where 57 and 64% of the respondents respectively reported experiencing a symptom consistent with HRI during a hot day. Headache was reported as the most common symptom suffered by participants, followed by muscle aches, elevated body temperature, difficulty breathing, dizziness, insomnia, and hot, dry skin [14]. Kearney et al.’s study in the USA also reported that the prevalence of having one HRI symptom was 72% and 27% among workers having three or more HRI symptoms [21]. Heat illness was higher than the rates reported by multiple other studies; in Uejio et al.’s study, in the USA, about 20% of workers reported experiencing one or more heat-related symptoms during the previous summer [1]. About a third of outdoor workers reported heat illness while working outside in another US study [22]. A study by Han et al. in China reported that only 11.6% of the participants had experienced heat-related illnesses in hot weather [9]. In another study in Italy, the percentage of HRI cases who required first aid or medical intervention was 10.49% [15].

The differences in the rates of heat-related illness in these studies demonstrated that intense and prolonged occupational exposure to elevated temperatures and working under direct sunlight is directly associated with the frequency of adverse health effects, such as dehydration and spasms, increased fatigue, and reduced productivity. This draws attention to the defective measures in intervention and defects in preventive measures specifically designed for severe hot climates during the warm season. Emphasizing administrative controls, particularly educating field supervisors and workers on how to avoid and recognize HRI, should be a priority.

More than half of the solid waste disposal workers reported that wearing thicker clothes does not help in maintaining a low body temperature. Similar to these results, Arcury et al. [21] and Kearney et al. [22] reported that outdoor workers wearing hats or dry clothing were less likely to experience HRI symptoms. Clothing can inhibit or prevent sweat evaporation and convective cooling [23]. Generally, heat exchange is hampered significantly by clothing that is heavier or less permeable [24]. Some types of clothing can prevent this mechanism from working, even in cool ambient conditions, by blocking the flow of cool, dry air over exposed skin [25]. Impermeable coveralls, for example, can prevent sweat from evaporating, and clothes and personal protection equipment (PPE) can generate microenvironments that retain heat close to the skin. Adjustments to exposure limits and work-rest regimens are necessary, based on the type of clothes and the job’s physical demands. Employers must be aware of this and take the necessary actions to remedy adverse workplace condition s[26].

Our results showed a deficiency in workers’ knowledge regarding the effects of climate change on the environment and types of heat-related illnesses. Only about one-third had adequate knowledge about these topics. Evidence shows that outdoor workers’ awareness of occupational heat stress varied by country/region and industry [27]. Usually, workers in developing nations typically have lower knowledge of heat risk and awareness of climate change than workers in developed nations [28], with a few exceptions, such as in Ghana [27] and in a USA study, in which outdoor worker awareness was only (8%) [29].

Compared with a study from Australia, Australian outdoor workers were almost twice as concerned about heat exposure as the present study’s participants [16] Our study’s relatively low awareness of workplace heat exposure may reflect the cultural and demographic differences between the two countries.

On the other hand, about two-thirds of the studied workers had adequate knowledge about symptoms of heat exhaustion, the symptoms of heat stroke, and that fainting and collapse could be due to heat-related illnesses. Published studies have consistently found that most workers in developing and developed countries have a good knowledge of heat illnesses [30, 31].

Evidence from the USA [29], India [32], South Africa [33], and Australia [34] showed that outdoor workers had a good knowledge of the symptoms and severe outcomes of excessive heat exposure. While physicians sometimes find difficulty in recognizing the early stages of [35, 36], workers often exhibit a good knowledge of symptoms and possible outcomes of excessive heat exposure, particularly in high-risk settings [16, 37].

Unsurprisingly, redesigning workplaces to avoid or minimize heat exposure among workers may sometimes be problematic or vastly exceed the available resources. However, many available heat management protocols, standards, and guidelines exist. Following these guidelines helps to lower or even avoid heat-related illnesses.

Our results showed that most participants had a deficiency in preventive measures (e.g., keeping hydrated, wearing light-colored breathable clothes, and stopping work). Keeping hydrated is vital to prevent heat stroke; however, cool drinking water may be unavailable during all working times. The unavailability of safe and cool drinking water in the workplace has been reported in different studies from Australia and the USA [16, 17].

The most prevalent work-related heat adaptation in our study was drinking plenty of fluids. This lower percentage of drinking water as a method of protection from heat-related illness is similar to the study from Moda et al. in which increased hydration was identified as a less common (43–58%) strategy used by the general public [38]. The percentage of drinking water as a method of protection was less than the rates reported in other studies where outdoor workers protect themselves from the heat by drinking plenty of liquids (85% and 90%), followed by wearing a hat and seeking shade (46% and 40%) [1, 15].

US federal guidelines recommend that outdoor workers drink ~ 0.25 L of cold (< 15 °C) water every 15 to 20 min [26]. When workers engage in moderate physical activity in the heat, sports drinks with balanced electrolytes should be consumed if sweating continues for several hours, and alcohol and beverages with a lot of sugar or caffeine should be avoided. Generally, six cups of liquids each hour is the maximum recommended consumption [19].

According to the OSHA guidelines, workers should rest for 15 min after each hour of work when White bulb globe temperature (WBGT) exceeds 26 °C, 30 min after each hour of work when WBGT reaches 28 °C, and 45 min of rest after each hour of work when WBGT reaches 30 °C [39].

Heat prevention strategies mainly include regulations, administrative controls, and engineering modifications. Currently, there are systematic technical guidelines and manuals in place for heat stress monitoring, risk assessment, control, and prevention, such as the ISO (International Organization for Standardization) heat indices, ACGIH (American Conference of Governmental Industrial Hygienists) guidelines, and NIOSH (US National Institute for Occupational Safety and Health) guidelines. The core elements of all heat-related illness prevention programs such as CDC, NIOSH, and OSHA are as follows: consuming adequate fluids, working shorter shifts, taking frequent breaks, quickly identifying any heat illness symptoms, engineering controls such as air conditioning, cooled air, and increasing airflow, changing workloads and schedules, and providing training to all supervisors and workers about heat-related symptoms and first aid [39, 40].

Evidence has shown that outdoor workers’ knowledge and awareness of HRI are affected by factors such as level of education, age, culture, country, duration of employment, and the local occupational safety management system [30] Usually, outdoor workers in developing countries had lower climate change awareness and adaptation behavior levels than workers in developed countries [31]. Our study’s analysis of the relationship between age and the total scores for knowledge and adaptation behavior showed that older workers were less aware of heat exposure than younger workers. Occupational HRI has been reported to occur more in younger workers, particularly workers who generate metabolic heat from heavy physical labor in hot environments [1]. The increased risk for younger workers (under 25 years old) could be caused by the more arduous tasks and physical activity experienced by workers in this age group. Also, younger workers are more likely to undertake physically demanding tasks in the workplace. Moreover, they often need more safety training or have fewer skills than older workers [38]. Some studies also observed that younger workers might be less likely to recognize the risk of heat exposure and show low compliance with preventive measures [39]. Older workers tend to choose duties with less exposure to direct sunlight and extreme heat. They have more power and authority than younger workers in choosing tasks in the workplace [40]. This highlights the need to prioritize improving the HRI and safety awareness of older solid waste disposal workers.

Interestingly, our results showed that knowledge and adaptation behavior of workers were significantly associated with educational level, which is inconsistent with an Italian study by Ricco et al, which revealed that higher educational achievements were positive predictors for higher workers’ awareness ,[41] underlining the substantial impact of appropriate information and education of workers in the process of building up appropriate awareness towards health risks.

### Study limitations

This study has some limitations that should be mentioned. Firstly, to the author’s knowledge, this is the first study to discuss climate change and HRI among solid waste disposal workers in Egypt, so there is a deficiency in studies to compare the situation in different regions in Egypt. Secondly, bias in recall and self-reporting inevitably exists in a cross-sectional observational study. However, we have taken measures such as piloting the questionnaire and shortening the recall period to minimize its impact.

## Conclusion

Solid waste disposal workers were at high risk of HRI due to their low levels of knowledge and adaptation behavior regarding HRI. Age and education were the most significant predictors of low levels of knowledge and adaptation behavior. Educational health programs that can guide workers to follow healthy behaviors and prevent HRI are recommended. Heat stress training is essential for increasing knowledge and adaptation behavior among solid waste disposal workers. Increasing the number of research studies directed at this vital sector, especially in hot climate countries like Egypt, is fundamentally an urgent need.

## Data Availability

The datasets used and analyzed during the current study are available from the corresponding author upon reasonable request.

## Abbreviations

HRI: Heat-related illness
RCPs: Representative concentration pathways
OSHA: Occupational safety and health administration
NIOSH: National Institute of Occupational Safety and Health
PPE: Personal protective equipment
WBGT: White bulb globe temperature
